# A feasibility and safety trial investigating a device for swift and standardized median laparotomy closure

**DOI:** 10.1007/s10029-025-03378-9

**Published:** 2025-06-03

**Authors:** Gabriel Börner, Lena Toft, Peder Rogmark, Marcus Edelhamre

**Affiliations:** 1 Department of Clinical Sciences Lund, Lund University, Lund, Sweden; 2 Department of Surgery, Helsingborg Hospital, Helsingborg, Sweden; 3 Department of Surgery, Skåne University Hospital Malmö, Malmö, Sweden; 4 Department of Clinical Sciences Malmö, Lund University, Malmö, Sweden

**Keywords:** Incisional hernia prevention, Suturing technique, Innovation, Abdominal wall closure, Laparotomy

## Abstract

**Purpose:**

Abdominal wall complications can be reduced by adhering to guidelines for midline laparotomy closure. However, implementation of guidelines can be challenging. To address this issue, a laparotomy closure device for swift and standardized abdominal closure was developed. The study evaluated the quality of the suture, safety, and speed of the device in a clinical setting.

**Methods:**

A prospective, one-armed investigation was carried out. Five surgeons participated in the study. The introduction to the device involved reading the user instructions and unsupervised dry lab training. Thirty-eight patients with colorectal disease, selected for laparotomy, were recruited. The primary endpoint was the proportion of patients that received a fascial closure with a suture-length to wound-length (SL/WL) ratio ≥ 4. Secondary endpoints included suturing time, glove puncture rate, wound infection (SSI), burst abdomen, and other adverse events. Follow-up included physical examination during hospital stay and postoperative visit and chart review six weeks postoperatively.

**Results:**

All patients achieved the primary endpoint SL/WL ratio ≥ 4. The mean suturing time was 10.5 min, while the mean net closure time (NCT) was 7.4 min. The shortest NCT recorded was 2.2 min. Net mean closure speed was 27 s/cm. There were no glove punctures. One case of SSI was reported, and no burst abdomen was detected. The learning curve stabilized after the third fascial closure.

**Conclusion:**

The SutureTOOL is a promising device for clinical application. It is perceived as safe, user-friendly, and fast, yielding a standardized laparotomy closure with a brief learning curve. The next steps involve a multi-center randomized trial to evaluate the potential impact of SutureTOOL on short- and long-term complications related to abdominal wall closure.

## Introduction

Laparotomy is tied to abdominal wall complications such as surgical site infection (SSI), wound dehiscence, and incisional hernia (IH) formation [1–3]. These complications impact the length of stay, antibiotic treatment, cost of wound care, and more importantly, the patient’s quality of life [1, 4]. Only 23% of patients with postoperative complications timely received the necessary adjuvant chemotherapy, a factor that can strongly affect survival [5].

While many surgical procedures utilize minimally invasive techniques (MIS), a significant proportion still necessitate open access. This includes debulking surgery, rapid bleeding control in trauma patients, and procedures for bowel perforation or obstruction [6–8]. One-third of open abdominal cases come from Caesarean sections. The global rates of Caesarean sections are projected to rise from 7% in 1990 to 29% by 2030 [9].

Additionally, the conversion rates from MIS to open surgery in bowel procedures range from 8 to 24% [10, 11]. The extraction site can exceed 10 cm, which essentially equates to a laparotomy, and is susceptible to complications such as SSIs (16.7%) and IH formation (12.6%) [12, 13].

Several clinical trials demonstrate that the technique of fascia closure significantly influences complications [14–16]. The fascia should be approximated using a suture-length to wound-length (SL/WL) ratio of ≥ 4, achieved through small-bites (5–8 mm fascial bites with a step-interval of 5 mm). This technique should employ a continuous suture line using a slowly absorbable suture [17]. Despite these recommendations being advocated in both elective and emergency surgery guidelines, the potential to reduce abdominal wall complications has received low attention [17, 18]. According to surveys performed with surgeons in Canada, The Netherlands and UK, only one fourth of surgeons utilize the small-bites technique [19–21]. Reasons for not adopting small-bites include a lack of familiarity with the methods needed to execute correctly (25%) and the perception that it takes too long (13%) [22]. Among surgeons who have received training and claim to apply this technique, only 31% manage to do so in actual clinical practice [23].

Suturing carries an inherent risk of sharp injury and exposure to blood-borne agents, with an accompanying issue of underreporting [24]. Over 50% of intraoperative sharp injuries are attributable to surgical needles, and the risk of a sharp injury increases by 22% per hour [25]. Most intraoperative sharp injuries occur during the closure of laparotomy wounds [26].

To address these clinical needs, a device for quick and standardized abdominal wall closure has been developed. Pre-clinical studies have demonstrated that the device can achieve an SL/WL ≥ 4 in 95–98% of cases, with a closure time that is 30% shorter compared to the traditional manual needle driver suturing technique [27, 28]. In addition, a glove test revealed no punctures following device suturing [28].

This study aimed to perform a safety and performance assessment of the device in the clinical setting.

## Method

### Trial design

The study was a prospective, single-centre, one-armed investigation of clinical performance and safety, assessing a device for laparotomy closure. The protocol was published on clinicaltrials.gov (ID NCT05695157) before study initiation.

### Participating surgeons

Five surgeons were invited to participate in the study. All surgeons were specialists and had been affiliated to the institution colorectal team for at least 2–8 years. Participating surgeons had no previous experience with the investigational device. One months before study start, surgeons were provided with a kit including one device, one printed instruction for use, two sutures, a forceps and a 30 × 30 cm large wooden model. The model was framed with fabric with a 20 cm long cut resembling an abdominal incision. Surgeons were instructed to read the instructions for use and practise until they felt comfortable using the device. No supervision or follow-up was performed. No examination was performed prior to clinical use and surgeons received no intraoperative proctoring.

### Study population

Patients aged 18 years and older, who were selected to undergo elective open surgery through midline laparotomy for colorectal disease and could fully comprehend the nature and purpose of the investigation, were invited to participate. All participants signed an informed consent form. Exclusion criteria included a prior midline incision or current midline hernia, pregnancy, clinical findings that interfere with the objectives of the investigation, collagen disease, disseminated disease, or a life expectancy of less than one year. Data on sex, age, height and weight, patient comorbidities, the indication for surgery, the operation performed, and the American Society of Anesthesiologists physical status classification (ASA) were collected.

### Outcomes

The primary endpoint was the proportion of patients with SL/WL ≥ 4. SL/WL was calculated by dividing the length of the suture used by the length of the laparotomy wound after closure.

Secondary endpoints included stitch-count, the number of sutures used, time taken to close the laparotomy, and a self-evaluation of the device using a visual analogue scale (VAS). Other intraoperative endpoints were incision not aligned with the midline, exposure of the rectus muscle, thickness of subcutaneous fat, glove puncture rate, re-operation, and unscheduled post-surgery visits. Safety endpoints comprised adverse events, SSI, and burst abdomen defined as post-operative separation of the abdominal musculo-aponeurotic layer.

### Accessory outcomes

The study protocol involved measuring the length of each suture, and the extra time required for these measurements was recorded and extracted to analyze the net closure time (NCT) – both generally, and specifically for closures performed after the learning curve was surmounted.

The mean stitch length was computed by dividing the total suture-length by the stitch-count.

**Fig. 1:**
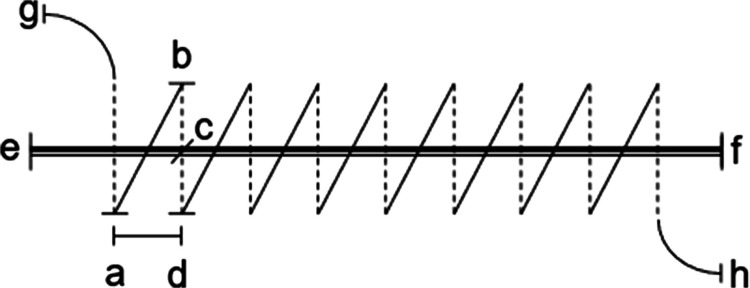
Figure shows a sequence of stitches in a continuous suture line. *abd* is one complete stitch, stitch-length. ad is the distance between two stitches, step interval. *bc* is bite-size. *ef* is wound-length (WL) and *gh* is the suture-length (SL) deployed in the wound for the laparotomy closure

Bite-size, *bs*, was calculated with the formula $bs=\frac{{sl}^2-{wl}^2}{4\cdot sc\;\cdot sl}$ where *sl* is suture-length, *wl* is wound-length and *sc* is stitch-count, assuming that the individual stitches form a right-angled triangle (Fig. 1). Stitch-time was calculated by dividing incision closure time with stitch-count for each incision closure.

**Fig. 2:**
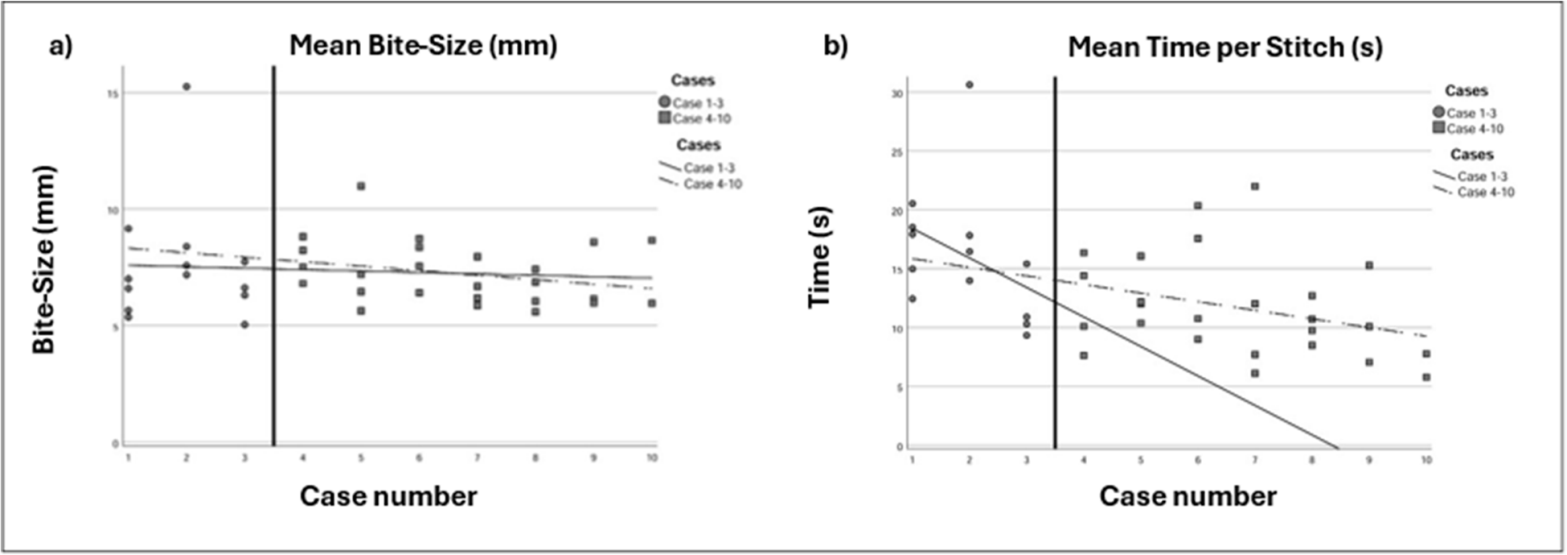
Figure shows all data points for a) bite-size (n = 38) and b) time per stitch (n = 38), for consecutive laparotomy closures (surgeons performed 1, 8, 9, 10 and 10 individual cases). Lines follow mean values. Straight lines follow cases 1–3 and dashed lines follow cases 4–10. mm, millimetre. s, seconds

A previous study with the investigational device indicated that the suture time stabilized after three incision closures [28]. To assess the learning curve piecewise linear regressions were performed for cases 1–3 and 4–10 respectively for mean bites-size and mean-time per stitch according to Fig. 2.

### Investigational device

**Fig. 3:**
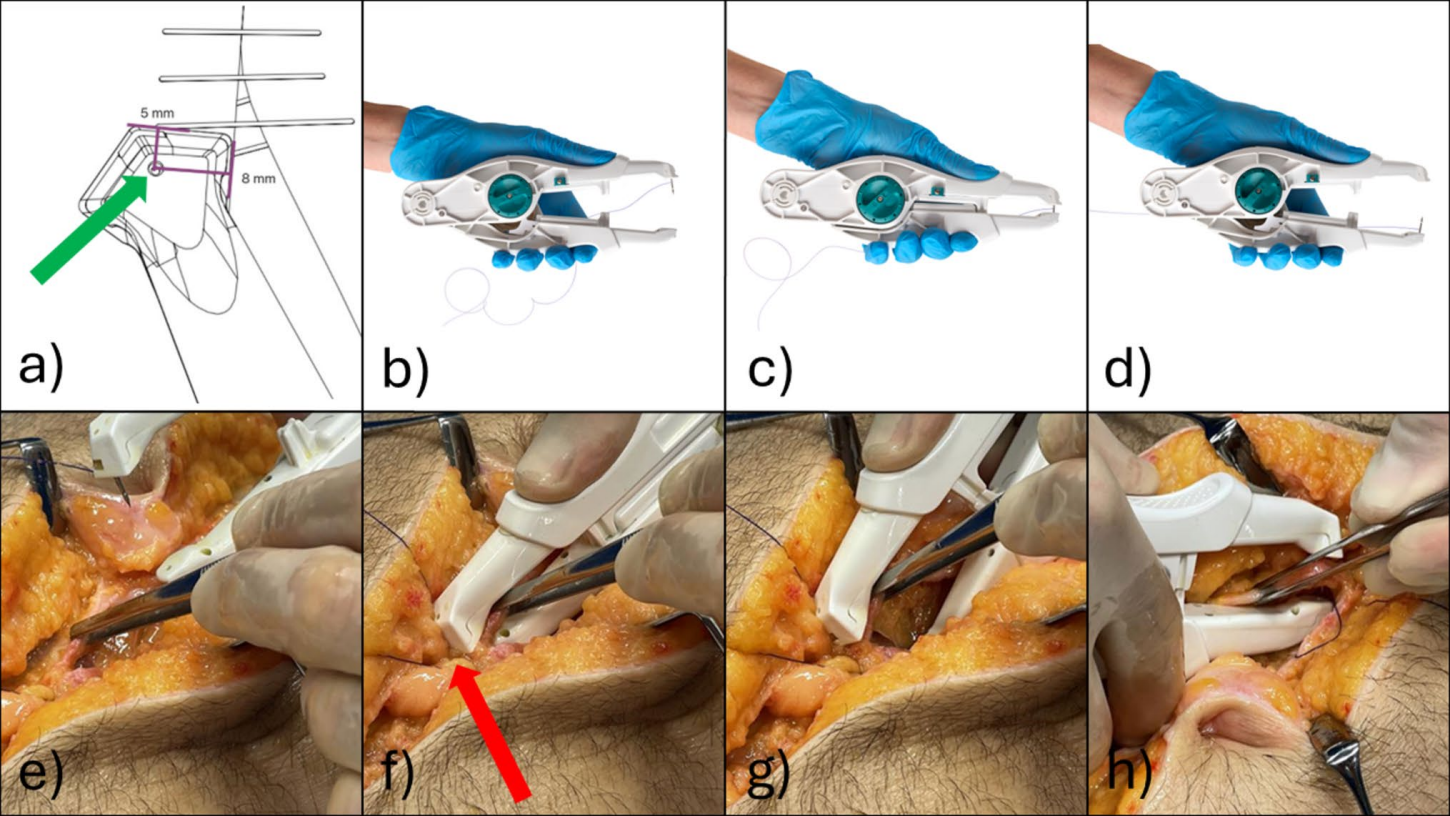
The figure shows the handling of the investigational device. **a**) Figure shows the front of the device's upper arm from above. The device has a guide at the front of the upper arm that facilitates small-bites placement. The guide is positioned adjacent to the previous stitch and with the lateral side towards the incision. Distance from the needle hole (green arrow) to the front of the guide is 5 mm and distance from the needle hole to the side of the guide is 8 mm. **b**) The device with a double-pointed needle attached in the upper arm. **c**) When the device is compressed the needle is transferred to the lower arm. **d**) Needle attached in the lower arm **e**) The forceps grab the contralateral edge of the midline aponeurosis. **f**) The guide (red arrow) is directed to the previous stitch. **g**) The lower arm of the device is released and the suture thread is pulled through the aponeurosis. **h**) The device is moved to the ipsilateral side of the incision to complete the stitch

The SutureTOOL (Suturion, Lund, Sweden) is a sterile, single-use, handheld, mechanical laparotomy closure device equipped with a double-pointed needle. The study was performed prior to market approval. This needle features a centrally attached suture thread of 130 cm long polydioxanone 2/0. The device includes a guide that enables small-bite stitch placement, specifically 5–8 mm fascial bite-size and a step-interval of less than 5 mm (Fig. 3a). The first author, in collaboration with Lund University, Lund, Sweden, was involved in its development.

### Technique of laparotomy closure

The intervention was restricted to the laparotomy closure portion at the end of the operation. The midline fascia was dissected free from subcutaneous fat one cm laterally to the incision. The length of the incision and subcutaneous fat layer was measured. Before the incision closure, both the surgeon and assistant switched to new surgical gloves. After this, the laparotomy closure was carried out with the device. The length of the suture was measured before use, as well as the remaining length after laparotomy closure. Closure time was documented from the completion of the first knot to the last stitch. Patients underwent the colorectal surgical procedure according to the local protocol, and the skin was adapted with skin staplers.

### Glove puncture test

The gloves of the surgeon and theatre nurse were collected after each laparotomy closure and labelled for identification. For the assessment, a filling tube capable of holding over 1000 ml of water was vertically placed in a test tube holder. The glove was attached to the lower opening of the tube and filled with 1000 ml of water. The glove was deemed intact if no leak was detected after 2 min.

### Follow-up

The research nurse, who is also a qualified stoma nurse, conducted the follow-up, which included a physical assessment and a chart review of SSI, burst abdomen, and other adverse events upon discharge and at a scheduled visit between 4–6 weeks. Unscheduled visits were identified through chart reviews at 6 weeks. Wound assessment was performed according to Center for Disease Control—Surgical Site Infection Criteria [29].

### Coordination, monitoring and data collection

The finalisation of the study protocol, study documentation, and primary data analysis were performed by a clinical research organisation (CRO) (CROSS Research S.A., Arzo, Switzerland). A research nurse conducted data collection in the operating theatre and during follow-ups, entering data in a paper-based case report form (CRF). This CRF was subsequently sent to the CRO and transferred into an electronic database for analysis. The study was externally monitored by a third part (Clinical Trial Consultants, Stockholm, Sweden). It should be noted that no part of the intervention was blinded.

### Statistical analysis

The statistical analysis was conducted using SAS® version 9.3 (TS1M1) for Windows®, or a newer version. The calculation of the sample size was based on a 70% success rate in SL/WL of 4, which is observed in common clinical practices, paired with the assumption that a SL/WL of 4 would be achieved in 83% of cases involving device-assisted laparotomy closure [28, 30]. Analysis of accessory outcomes was performed with IBM SPSS Statistics v26.

### Ethical considerations

The clinical investigation was conducted following the general principles of ISO 14155:2020(E) – Clinical Investigation of Medical Devices for Human Subjects – GCP, as of July 2020, the European Regulation 2017/745 on Medical Devices, and MDCG 2020–10/1 Rev. 1. Study approval, which included ethical approval, was obtained from the Swedish Medical Products Agency (CIV-ID 22–09–040607).

## Results

The screening process included 41 patients for inclusion. However, two patients opted not to participate, and one patient was excluded because the study staff was not available during the scheduled surgery time. Baseline and surgical procedure data can be found in Table 1.

**Table 1:** Demographic and procedure data on 38 patients undergoing elective median laparotomy due to colorectal disease

Demographic and procedure data	Total (n = 38)
Sex ratio (M:F)	19:19
Age (years)	74 (9)
ASA	
I	2
II	25
III	11
IV	0
Comorbidities	
Diabetes mellitus	12
Hypertension	20
Respiratory disorders	5
Anemia	3
Height (cm)	170 (9)
BMI (kg/m^2^)	27.4 (4.6)
Indication for surgery	
Colon or rectal cancer	36
Benign disease	2
Performed procedure	
Right hemicolectomy	24
Left hemicolectomy	2
Sigmoid resection	6
Rectum resection	3
Other	3
Stoma formation	5

Values are n, number of patients, unless otherwise indicated. ASA, American Society of Anesthesiologists physical status classification. BMI, body mass index. SD, standard deviation

### Intraoperative outcomes

In all patients, a SL/WL ≥ 4 was achieved on the first attempt, thus the proportion of patients with SL/WL ≥ 4 was 100%. The mean thickness of subcutaneous fat was 36.9 mm (standard deviation (SD) 12.5).

All incisions were along the midline, but the impact on rectus muscle exposure was excluded from the analysis due to a misunderstanding.

The gloves of surgeons and assistants (n = 152) were tested and no leaks were detected. Further secondary intraoperative endpoints are reported in Table 2.

**Table 2:** Intraoperative outcomes

Outcome	All patients (n = 38)	After learning curve (n = 25)
Patients with SL/WL ≥ 4	38	25
SL/WL ratio	7.6 (2.3)	7.2 (2.1)
Wound length (cm)	16.1 (3.6)	16.2 (2.1)
Stitch count	41.4 (15.1)	39.8 (16.3)
Number of sutures used	1.6 (0.6)	1.5 (0.7)
Incision closure time (min)	10.4 (5.1)	9.0 (5.0)
NCT/incision length (s/cm)	31.6 (12.2)	26.7 (10.6)
NCT (min)	8.6 (3.7)	7.4 (3.5)
NCT (min), Median (range)	8.1 (2.2–23.6)	6.5 (2.2–23.6)
Stitch-length (cm)	3.0 (0.7)	3.0 (0.5)
Bite-size (mm)	7 (2)	7 (1)

Values are mean, SD unless otherwise indicated. SL/WL, suture length/wound length. SD, standard deviation. NCT, net closure time. min, minutes. s, seconds. Bite size is calculated by assuming each stitch forms a right-angle triangle

### Surgeons

**Fig. 4:**
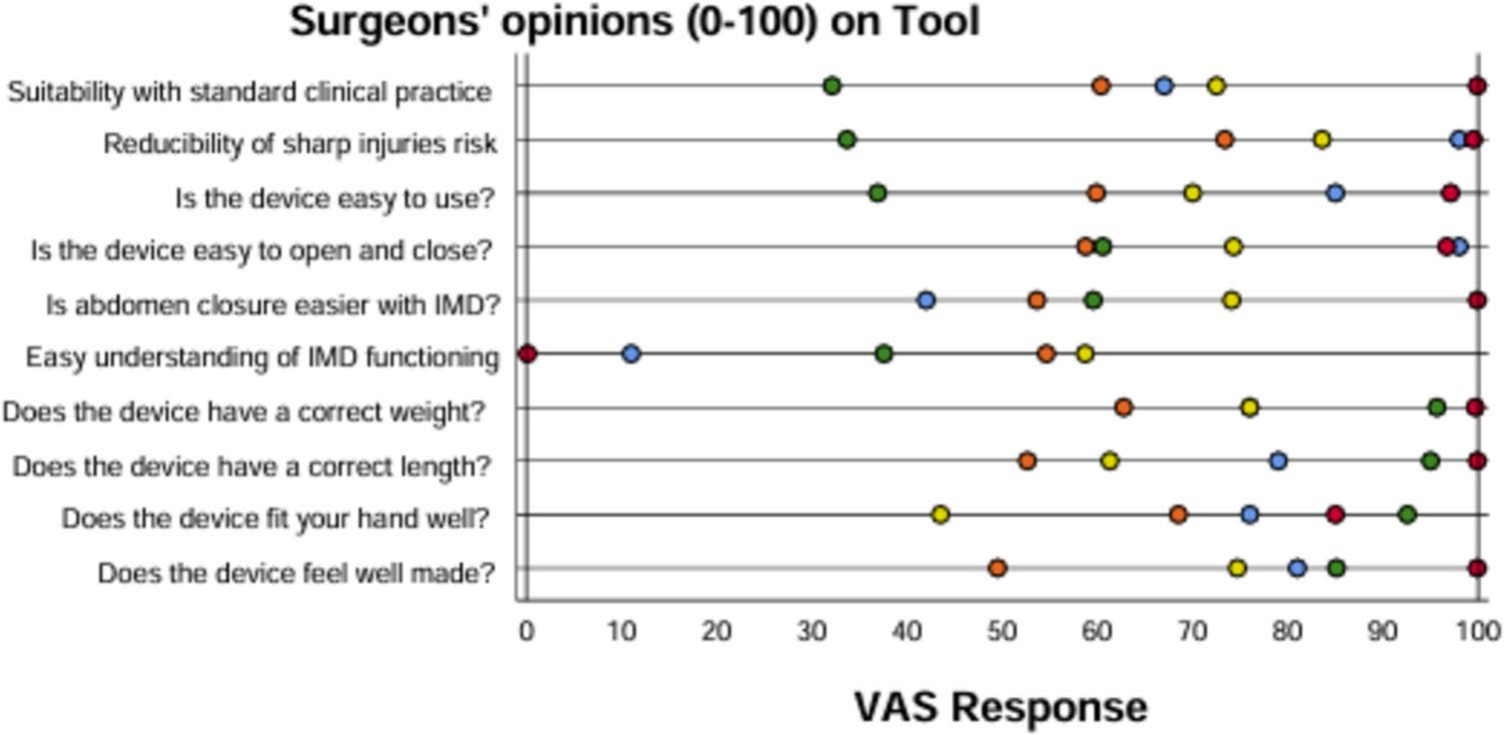
Surgeons'opinion about the investigational device. Each coloured dot indicates the individual surgeon's mean response (1–10 responses) after laparotomy closure (n = 38). IMD, investigational Medtech device

The study involved the participation of three female and two male surgeons. They performed one, eight, nine, ten, and ten closures respectively. After each laparotomy closure, the surgeons completed the VAS evaluation form (Fig. 4). The operating surgeon completed the questionnaire after each patient, yielding between 1 to 10 completed questionnaires from each of the five participating surgeons. The mean response of each surgeon is presented as colour-separated dots on individual statements in the figure.

### Follow-up

All patients completed the 6-week follow-up. One patient had a superficial incisional SSI treated with negative pressure treatment and antibiotics which resolved after 4 weeks. During the final visit, an additional patient was diagnosed with a superficial SSI that resolved by wound care. No patients suffered from a burst abdomen, and there were no other abdominal wall-related complications detected. One patient was re-operated due to suspicion of an anastomotic leak. Two patients received negative pressure treatment due to seroma formation and re-operation respectively. Unscheduled visits were made postoperatively by three patients to their general practitioners and four underwent emergency room visits for wound unrelated issues. Two patients did unscheduled outpatient clinic visits for wound management. The number of post-surgery visits and re-operations fell in line with normal clinical practice and did not raise any safety concerns.

### Adverse events

No patients discontinued the study due to adverse events.

### Accessory analysis

The overall mean bite-size was 7 mm (SD 2.0), and the mean bite-size of consecutive closures are shown in Fig. 2a.

The mean NCT for all closures was 8.6 (SD 3.7) min, and the mean NCT for consecutive closures 4–10 was 7.4 (SD 3.5) min.

Regression coefficient for bites-size cases 1–3 and 4–10 respectively was −0.061 and – 0.192 and this difference was −0.131 (95% CI −1.469 – 1.208). There was a change in regression coefficient for stitch-time between cases 1–3 and 4–10 respectively from −2.505 to −0.730, but this difference 1.774 did not receive significance level (95% CI −1.490- 5.039) (Fig. 2a).

## Discussion

The risk of complications in abdominal wall closure can be reduced if surgeons adhere to the recommendations of the European and American hernia societies, as well as the World Society of Emergency Surgery. These guidelines advocate for abdominal incision closure with a SL/WL of 4 or more, achieved through the use of small-bites [17, 18].

In contrast to clinical practice, all patients in this study, which evaluated device-assisted laparotomy closure, received a SL/WL ≥ 4, thereby meeting the requirements from the guidelines. Pereira-Rodríguez et al. reported a mere 31% success rate in achieving small-bites after formal training, while an audit of institutional practice by Williams et al. found that laparotomy closure with SL/WL ≥ 4 was achieved in 76% of cases, but only in 46% of emergent cases [23, 30]. In another study, Golling et al. demonstrated the difficulty in achieving high SL/WL with small-bites. After an initial failure, the study was restarted and, following training, instances of small-bites with SL/WL ≥ 4 increased to 87%. However, the study’s goal of a SL/WL > 6 was only achieved in 44% of cases [31].

The mean SL/WL in the current study was 7.3, which is higher compared to the 2015 STITCH trial (SL/WL of 5.0), and the 2009 Millbourn et al. trial (SL/WL of 5.7), both of which showed a reduction in IH formation when small-bites were applied [3, 14]. Although the optimal range for the SL/WL ratio for laparotomy closure has yet to be identified, there are indications that a ratio > 7:1 might be safe. Harlaar et al. demonstrated that the initial burst strength in a porcine abdominal wall model was higher with small-bites with an SL/WL of 6.9 (range 5.0–8.6) [32]. Furthermore, in a rodent model, Höer et al. showed that closures with an SL/WL ratio of 4:1 to 8:1 yielded the highest tensile strength after 14 days [33].

Each stitch length in a suture line depends on the interval between stitches, the size of each bite, and the pull tension on the suture thread. A study by Millbourn et al. demonstrated a correlation between stitch length, SSI, and IH formation [34]. With stitch lengths of less than 4 cm, the rates of SSI and IH formation were found to be 4% and 3% respectively, compared to 8% and 11% for stitch lengths of 4–4.9 cm, and 16% and 12% for stitch lengths greater than or equal to 5 cm. Thus, to achieve an SL/WL ratio greater than or equal to 4, the recommended practice is to use stitches shorter than 4 cm, with an individual bite-size of 5–8 mm, a method now referred to as “small-bites”. In our current study, the mean stitch length was 3 cm, with a mean bite-size of 7 mm – within the desired range of 5–8 mm. The consistency of the bite-size in consecutive incision closures throughout the study suggests that the investigational device can standardize the placement of small-bites (Fig. 2a).

Abdominal fascial closure using the small-bites method is a time-consuming task, generally ranging from 14 to 30 min in elective abdominal procedures, even when the surgeons are familiar with the closure technique [3, 35, 36]. The overall mean closure time in this study was 10.4 min, which included specific measurements of the length of the new suture whenever more than one suture was used. To better reflect common clinical practice, the first three closure times and any additional time spent measuring the suture were deducted in a separate analysis. This adjustment indicated a median NCT of 6.5 min for an average 16 cm long incision. In a survey about knowledge and attitudes toward hernia prevention, Fisher et al. found that one of the reasons for not using the small-bites closure technique was its time-consuming nature [22]. Numerous clinical studies have documented the time needed for manual laparotomy closure, finding that small-bites typically take approximately 30% longer than large-bites. However, the most rapid closure time in our study was just 2.2 min for a 12 cm incision, suggesting that the device may considerably reduce closure time and potentially lower barriers to using small-bites.

Clinical trials involving the small-bites closure technique advocate the dissection of subcutaneous fat to expose the midline and facilitate a precise incision at the intersecting aponeuroses of the three vertical abdominal muscles, also known as the linea alba [36, 37]. This is essential to avoid opening the rectus muscle compartments and to ensure that the small-bites closure only involves the fascial edges [38]. However, there have been concerns that undermining the subcutaneous fat could potentially increase the risk of postoperative seroma and subsequent SSI. Despite these concerns, Albertsmeier et al. and Wenzelberg et al. recorded superficial SSI rates of 3.3% and 7.5% respectively in the small-bites groups and found low rates of seroma formation. Therefore, their findings suggest that the dissection of the fascia to facilitate small-bites closure seems safe [36, 39].

Sharp injury is common in open surgery when surgeons manipulate the needle with their hands or during the passing of sharp instruments. Suture needles account for 77% of intraoperative sharp injuries and half of the glove punctures occur during laparotomy closure [26, 40]. The study tested all surgeon and assistant gloves and no punctures were detected which is in line with findings in a pre-clinical study showing no punctures in 90 gloves when the investigational device was used [28]. This is an important finding as the prevention of sharp injuries is crucial for surgeon and staff safety.

The learning curve for surgery can be defined as the “time taken and/or the number of procedures an average surgeon needs to be able to perform a procedure independently with a reasonable outcome” [41]. Tracking the progression of stitch-time while maintaining closure quality can serve as a measurement of this learning curve. Per the protocol, the surgeons involved in the study received only minimal training in using the device before its in vivo application. As all laparotomy closures in the study had a SL/WL ≥ 4 and the bite-size was deemed to be consistent, we based the learning curve on stitch-time. In a pre-clinical study, the device’s learning curve was found to stabilize after three incision closures [28]. In this study we compared mean stitch-time from surgeons first three cases with the following in a piece-wise linear regression model (Fig. 2b). The difference of 1.774 did not reach significance level, probably due to small sample size. A short learning curve is vital for clinical implementation, especially considering the cautious adoption of the manual small-bites closure technique within the surgical community.

Some weaknesses of the study need to be addressed. First, the two previous publications on the device included a questionnaire with ten statements evaluating surgeon opinions of the device presented on a VAS scale [27, 28]. In the previous presentations, which included a total of 25 participants answering one set of statements each, all statements received VAS scores above 8. The highest scores were given to “Function is easy to understand” (VAS 9.4) and “Tool facilitates adherence to ratio of 4:1” (VAS 9.3). In the present study, surgeons completed the questionnaire after each patient, yielding between 1 to 10 completed questionnaires from each of the five surgeons (Fig. 4). Interpretation was difficult, and comparison with previous opinion presentations was not possible. Responses exhibited high individual variation, but overall they seemed to lean towards the right, indicating a favourable opinion about the device. However, a significant finding was that “Easy understanding of the IMD (device) functioning” received the lowest scores. This issue needs to be addressed in the IFU and device training.

This first-in-man clinical trial had a limited sample size and a small number of participating surgeons to address the primary endpoint, the proportion of patients receiving a laparotomy closure with SL/WL ≥ 4. Common clinical outcomes in abdominal wall closure technique research, such as burst abdomen, SSI, and IH formation, were beyond the scope of this study. Although short-term clinical data was collected to monitor adverse events, the study was not sufficiently powered to analyze and compare these outcomes in detail. Nonetheless, no burst abdomen was detected, and only one clinically significant SSI and one seroma were observed.

This was the fourth time we did structured testing of the device with surgeons within a scientific protocol. Previous studies provided us with an appreciation of the learning curve why we decided to reduce training. Surgeons managed to close all incisions with the correct SL/WL ratio, but the minimal training and lack of intraoperative proctoring probably had negative impact on closure time and efficiency. The device seems intuitive and surgeons experienced in laparotomy closure can understand how it works. For introduction in standard clinical practise, training needs to be far more thorough, and intraoperative proctoring might be needed to secure correct use of the device.

The study reveals that participating surgeons realized a 100% SL/WL ≥ 4 ratio with small-bites, demonstrating a potential to reduce abdominal wall complications. Suturing was determined to be faster than conventional laparotomy closure, and no glove punctures were detected, thus posing no safety issues for the surgeon.

SutureTOOL have the potential to raise attention to abdominal wall closure and to impact surgeons closing habits. It would be interesting to assess surgeons’ opinion about abdominal wall closure technique across different surgical communities after market introduction of the SutureTOOL. To address the potential impact on abdominal wall related complications, a large-scale, randomized, multi-center trial would be ideal to track SutureTOOL impact on SSI, burst abdomen and one year incisional hernia formation.

In conclusion, SutureTOOL represents a promising advancement in laparotomy closure, potentially enhancing surgical practice by providing a faster, safer, and standardized approach.
